# Dilead(II) trimanganese(II) bis(hydrogenphosphate) bis(phosphate)

**DOI:** 10.1107/S1600536812033259

**Published:** 2012-07-28

**Authors:** Abderrazzak Assani, Mohamed Saadi, Mohammed Zriouil, Lahcen El Ammari

**Affiliations:** a Laboratoire de Chimie du Solide Appliquée, Faculté des Sciences, Université Mohammed V-Agdal, Avenue Ibn Battouta, BP 1014, Rabat, Morocco

## Abstract

The title compound, Pb_2_Mn_3_(HPO_4_)_2_(PO_4_)_2_, was synthesized by a hydro­thermal method. All atoms are in general positions except for one Mn atom which is located on an inversion center. The framework of the structure is built up from PO_4_ tetra­hedra and two types of MnO_6_ octa­hedra, one almost ideal and the other very distorted with one very long Mn—O bond [2.610 (4) Å compared an average of 2.161 Å for the other bonds]. The centrosymetric octa­hedron is linked to two distorted MnO_6_ octa­hedra by an edge common, forming infinite zigzag Mn_3_O_14_ chains running along the *b* axis. Adjacent chains are linked by PO_4_ and PO_3_(OH) tetra­hedra through vertices or by edge sharing, forming sheets perpendicular to [100]. The Pb^2+^ cations are sandwiched between the layers and ensure the cohesion of the crystal structure. O—H⋯O hydrogen bonding between the layers is also observed.

## Related literature

For properties of phosphates and their potential applications, see: Gao & Gao (2005 ▶); Viter & Nagornyi (2009 ▶); Clearfield (1988 ▶); Trad *et al.* (2010 ▶). For compounds with related structures, see: Assani *et al.* (2010 ▶, 2011*a* ▶,*b* ▶,*c* ▶, 2012 ▶); Effenberger (1999 ▶). For bond-valence analysis, see: Brown & Altermatt (1985 ▶).

## Experimental

### 

#### Crystal data

Pb_2_Mn_3_(HPO_4_)_2_(PO_4_)_2_
*M* *_r_* = 961.10
Monoclinic, 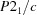

*a* = 7.9449 (2) Å
*b* = 8.8911 (2) Å
*c* = 9.5718 (3) Å
β = 100.917 (2)°
*V* = 663.90 (3) Å^3^
*Z* = 2
Mo *K*α radiation
μ = 28.63 mm^−1^
*T* = 296 K
0.18 × 0.12 × 0.08 mm

#### Data collection

Bruker X8 APEXII diffractometer
Absorption correction: multi-scan (*SADABS*; Sheldrick, 2003) ▶ *T*_min_ = 0.029, *T*_max_ = 0.117
8738 measured reflections
1225 independent reflections
1202 reflections with *I* > 2σ(*I*)
*R*_int_ = 0.044

#### Refinement

*R*[*F* ^2^ > 2σ(*F* ^2^)] = 0.020
*wR*(*F* ^2^) = 0.051
*S* = 1.10
1225 reflections
116 parameters
H-atom parameters constrained
Δρ_max_ = 1.37 e Å^−3^
Δρ_min_ = −2.03 e Å^−3^

### 

Data collection: *APEX2* (Bruker, 2005 ▶); cell refinement: *SAINT* (Bruker, 2005 ▶); data reduction: *SAINT*; program(s) used to solve structure: *SHELXS97* (Sheldrick, 2008 ▶); program(s) used to refine structure: *SHELXL97* (Sheldrick, 2008 ▶); molecular graphics: *ORTEP-3 for Windows* (Farrugia, 1997 ▶) and *DIAMOND* (Brandenburg, 2006 ▶); software used to prepare material for publication: *PLATON* (Spek, 2009 ▶) and *publCIF* (Westrip, 2010 ▶).

## Supplementary Material

Crystal structure: contains datablock(s) I, global. DOI: 10.1107/S1600536812033259/hp2043sup1.cif

Structure factors: contains datablock(s) I. DOI: 10.1107/S1600536812033259/hp2043Isup2.hkl

Additional supplementary materials:  crystallographic information; 3D view; checkCIF report

## Figures and Tables

**Table 1:** Hydrogen-bond geometry (Å, °)

*D*—H⋯*A*	*D*—H	H⋯*A*	*D*⋯*A*	*D*—H⋯*A*
O8—H8⋯O4	0.86	1.60	2.437 (5)	164
O8—H8⋯O1	0.86	2.76	3.393 (5)	132

**Fig. 1.**
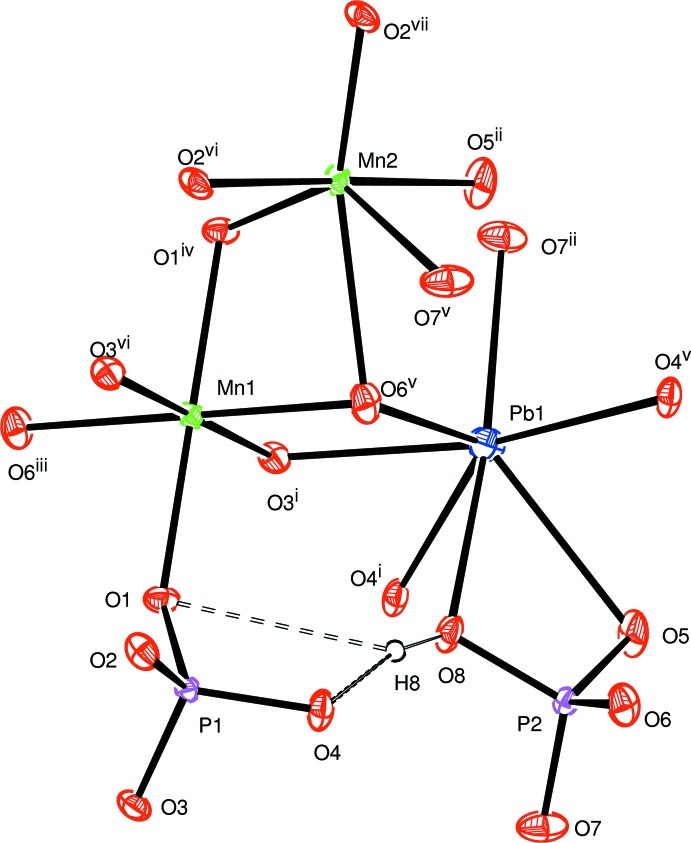
A partial three-dimensional plot of the crystal structure of the title compound. Displacement ellipsoids are drawn at the 50% probability level. Symmetry codes:(i) x, -y + 3/2, z + 1/2; (ii) -x + 1, -y + 1, -z + 1; (iii) -x + 1, y - 1/2, -z + 3/2; (iv) -x, y - 1/2, -z + 1/2; (v) x - 1, y, z; (vi) -x, -y + 1, -z + 1; (vii) x, -y + 1/2, z + 1/2; (viii) x, -y + 1/2, z - 1/2; (ix) -x, y + 1/2, -z + 1/2; (x) x, -y + 3/2, z - 1/2; (xi) -x + 1, y + 1/2, -z + 3/2; (xii) x + 1, y, z.

**Fig. 2.**
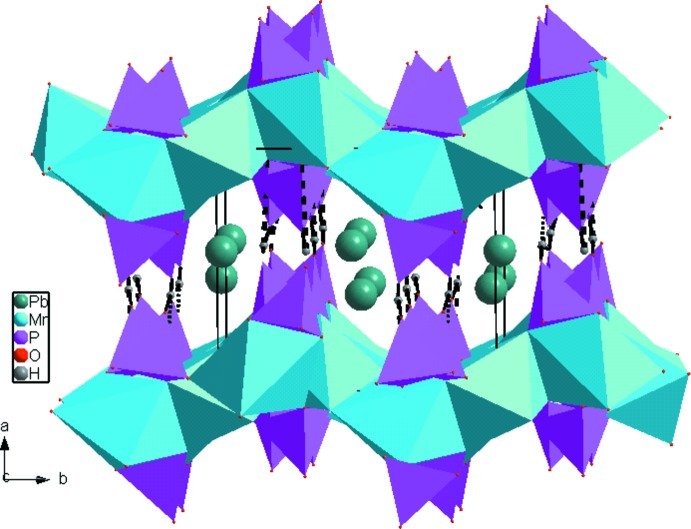
A three-dimensional polyhedral view of the crystal structure of the PbMn1.5(PO4)(HPO4), showing the stacking of layers along the a axis and the hydrogen bonding scheme (dashed lines).

## supplementary crystallographic information

### Comment

Owing to their remarkable variety of structures and to their outstanding potentialities in widespread applications such as catalysis (Viter & Nagornyi, 2009; Gao & Gao, 2005) and ion-exchangers (Clearfield, 1988) and in batteries performance (Trad *et al.*, (2010)), transition metal based phosphates have received great attention and still remains in the forefront of the developed scientific axes in our laboratory. Within this family of compounds, the resulting anionic frameworks, generally constructed from the alternation of PO_4_ tetrahedra connected to metal cations in different coordinate geometry MO_n_ (with n=4, 5 and 6), generate pores and channels offering suitable environment to accommodate different other cations. Accordingly, we have succeeded to isolate new silver metal based orthophosphates for instance, AgMg_3_(PO_4_)(HPO_4_)_2_ wich represent a new member of the well known alluaudite-like structure family (Assani *et al.* 2011*a*); silver (nickel or cobalt) phosphate, namely, Ag_2_*M*_3_(HPO_4_)(PO_4_)_2_ with *M*=Ni, Co (Assani *et al.* 2011*b*; Assani *et al.* 2011*c*). Furthermore, a special attention have been paid to the ternary system MO—*M*'O—P_2_O_5_ with *M*=Ba, Ca, Cd, Pb and Sr and *M*'= transition metals, Mg and Zn. Our recent investigation has allowed to the isolate the compounds Ni_2_Sr(PO_4_)_2_.2H_2_O (Assani *et al.* 2010) and Co_2_Pb(HPO_4_)(PO_4_)OH H_2_O (Assani *et al.* 2012).

Inline with the focus of our research, the present paper aims to develop the hydrothermal synthesis and the structural characterization of a new layered lead manganese orthophosphate, namely, PbMn_1.5_(PO_4_)(HPO_4_), which is characterized by Mn/P ratio =3/4, rarely encountered in the literature with the exception of some copper based orthophosphates, Pb_3_Cu_3_(PO_4_)_4_ and Sr_3_Cu_3_(PO_4_)_4_ (Effenberger 1999).

A partial three-dimensional plot of the crystal structure of PbMn_1.5_(PO_4_)(HPO_4_) is represented in Fig. 1. A l l atoms of this structure are in general positions, except one manganese Mn1 located in symmetry center (1) 2a (0 0 0; 0 1/2 1/2) of *P*2_1_/*c* space group. The network is built up from two different types of polyhedra more or less distorted, *viz*. PO_4_, HPO_4_ tetrahedra and Mn1O_6_ (1 symmetry), Mn2O_6_ octahedra. Moreover, the edge-sharing Mn1O_6_ and Mn2O_5_(OH) octahedra form an infinite zigzag chains ^1^∞ [Mn_3_O_14_] running parallel to [010], as shown in Fig. 2. Adjacent chains are connected by PO_4_ and HPO_4_ tetrahedra *via* vertices in the way to build layers parallel to (100). These layers are in turn linked by Pb^2+^ cations as shown in Fig.2. The strong hydrogen bonding between the layers is also involved in the stability of this structure (Fig. 2 and Table 2).

Bond valence sum calculations (Brown & Altermatt, 1985) for Pb1^2+^, Mn1^2+^, Mb2^2+^, P1^5+^ and P2^5+^ ions are as expected, *viz*. 1.82, 2.13, 1.97, 4.95 and 5.01 valence units, respectively. The values of the bond valence sums calculated for all oxygen atoms are between 1.93 and 2.05 except O4 and O8 which shown low values: 1.62 and 1.50 respectively. These atoms are considerably undersaturated and thus act as an acceptor with a very short H-bond (Table 2, Fig.1).

### Experimental

The crystals of the title compound is isolated from the hydrothermal treatment of the reaction mixture of lead oxide, metallic manganese and 85wt% phosphoric acid in a proportion corresponding to the molar ratio Pb:Mn:*P* = 1,5: 3:3.

The hydrothermal reaction was conducted in a 23 ml Teflon-lined autoclave, filled to 50% with distilled water and under autogeneous pressure at 483 K for twenty hours. After being filtered off, washed with deionized water and air dried, the reaction product consists of a light brown solid and colorless sheet shaped crystals corresponding to the title compound.

### Refinement

The O-bound H atoms were initially located in a difference map and refined with O—H distance restraints of 0.86 (1). In the last cycle they were refined in the riding model approximation with *U*_iso_(H) set to 1.2*U*_eq_(O). The highest peak and the deepest hole in the final Fourier map are at 0.62 Å and 0.67 Å, respectively, from Pb1. The not significants bonds and angles were removed from the CIF file.

### Crystal data

**Table:**

Pb_2_Mn_3_(HPO_4_)_2_(PO_4_)_2_	*F*(000) = 858
*M**_r_* = 961.10	*D*_x_ = 4.808 Mg m^−^^3^
Monoclinic, *P*2_1_/*c*	Mo *K*α radiation, λ = 0.71073 Å
Hall symbol: -P 2ybc	Cell parameters from 1225 reflections
*a* = 7.9449 (2) Å	θ = 2.6–25.4°
*b* = 8.8911 (2) Å	µ = 28.63 mm^−^^1^
*c* = 9.5718 (3) Å	*T* = 296 K
β = 100.917 (2)°	Prism, pink
*V* = 663.90 (3) Å^3^	0.18 × 0.12 × 0.08 mm
*Z* = 2	

### Data collection

**Table:**

Bruker X8 APEXII diffractometer	1225 independent reflections
Radiation source: fine-focus sealed tube	1202 reflections with *I* > 2σ(*I*)
Graphite monochromator	*R*_int_ = 0.044
φ and ω scans	θ_max_ = 25.4°, θ_min_ = 2.6°
Absorption correction: multi-scan (*SADABS*; Sheldrick, 2003)	*h* = −9→9
*T*_min_ = 0.029, *T*_max_ = 0.117	*k* = −10→10
8738 measured reflections	*l* = −11→11

### Refinement

**Table:**

Refinement on *F*^2^	Secondary atom site location: difference Fourier map
Least-squares matrix: full	Hydrogen site location: inferred from neighbouring sites
*R*[*F*^2^ > 2σ(*F*^2^)] = 0.020	H-atom parameters constrained
*wR*(*F*^2^) = 0.051	*w* = 1/[σ^2^(*F*_o_^2^) + (0.0232*P*)^2^ + 2.912*P*] where *P* = (*F*_o_^2^ + 2*F*_c_^2^)/3
*S* = 1.10	(Δ/σ)_max_ = 0.001
1225 reflections	Δρ_max_ = 1.37 e Å^−^^3^
116 parameters	Δρ_min_ = −2.03 e Å^−^^3^
0 restraints	Extinction correction: *SHELXL97* (Sheldrick, 2008), Fc^*^=kFc[1+0.001xFc^2^λ^3^/sin(2θ)]^-1/4^
Primary atom site location: structure-invariant direct methods	Extinction coefficient: 0.0091 (4)

### Special details

**Table:**

Geometry. All s.u.'s (except the s.u. in the dihedral angle between two l.s. planes) are estimated using the full covariance matrix. The cell s.u.'s are taken into account individually in the estimation of s.u.'s in distances, angles and torsion angles; correlations between s.u.'s in cell parameters are only used when they are defined by crystal symmetry. An approximate (isotropic) treatment of cell s.u.'s is used for estimating s.u.'s involving l.s. planes.
Refinement. Refinement of *F*^2^ against all reflections. The weighted *R*-factor *wR* and goodness of fit *S* are based on *F*^2^, conventional *R*-factors *R* are based on *F*, with *F* set to zero for negative *F*^2^. The threshold expression of *F*^2^ > 2σ(*F*^2^) is used only for calculating *R*-factors(gt) *etc*. and is not relevant to the choice of reflections for refinement. *R*-factors based on *F*^2^ are statistically about twice as large as those based on *F*, and *R*- factors based on all data will be even larger.

### Fractional atomic coordinates and isotropic or equivalent isotropic displacement parameters (Å^2^)

**Table:**

	*x*	*y*	*z*	*U*_iso_*/*U*_eq_	
Pb1	0.41097 (3)	0.52400 (2)	0.75343 (2)	0.01734 (13)	
Mn1	0.0000	0.5000	0.5000	0.0091 (2)	
Mn2	0.10770 (9)	0.13892 (8)	0.59303 (7)	0.00981 (19)	
P1	0.14019 (16)	0.70441 (14)	0.23105 (12)	0.0076 (3)	
P2	0.65619 (16)	0.70724 (14)	0.56583 (12)	0.0087 (3)	
O1	0.0496 (5)	0.6808 (4)	0.3570 (4)	0.0123 (7)	
O2	0.0740 (5)	0.5969 (4)	0.1080 (3)	0.0122 (7)	
O3	0.1156 (5)	0.8690 (4)	0.1828 (4)	0.0127 (7)	
O4	0.3370 (5)	0.6828 (4)	0.2816 (4)	0.0133 (7)	
O5	0.7064 (5)	0.6754 (5)	0.7236 (4)	0.0208 (9)	
O6	0.7536 (5)	0.6118 (4)	0.4742 (4)	0.0152 (8)	
O7	0.6777 (5)	0.8718 (4)	0.5276 (4)	0.0212 (9)	
O8	0.4617 (5)	0.6642 (4)	0.5341 (4)	0.0146 (7)	
H8	0.4002	0.6667	0.4499	0.022*	

### Atomic displacement parameters (Å^2^)

**Table:**

	*U*^11^	*U*^22^	*U*^33^	*U*^12^	*U*^13^	*U*^23^
Pb1	0.01783 (17)	0.01676 (17)	0.01923 (17)	0.00271 (7)	0.00812 (10)	0.00201 (7)
Mn1	0.0108 (6)	0.0084 (5)	0.0080 (5)	0.0006 (4)	0.0018 (4)	0.0002 (4)
Mn2	0.0096 (4)	0.0107 (4)	0.0082 (4)	0.0003 (3)	−0.0008 (3)	−0.0006 (3)
P1	0.0080 (6)	0.0090 (6)	0.0055 (6)	−0.0004 (5)	0.0003 (5)	0.0003 (4)
P2	0.0080 (6)	0.0111 (6)	0.0067 (6)	−0.0007 (5)	0.0009 (5)	−0.0005 (4)
O1	0.0154 (19)	0.0110 (17)	0.0120 (17)	−0.0005 (14)	0.0068 (14)	0.0016 (13)
O2	0.0154 (19)	0.0111 (17)	0.0076 (16)	0.0002 (14)	−0.0045 (14)	−0.0020 (13)
O3	0.0154 (19)	0.0099 (17)	0.0120 (17)	0.0005 (14)	0.0008 (14)	0.0026 (14)
O4	0.0083 (18)	0.0234 (19)	0.0078 (16)	0.0002 (14)	0.0004 (13)	−0.0002 (14)
O5	0.015 (2)	0.038 (2)	0.0076 (18)	0.0013 (17)	−0.0021 (14)	0.0035 (16)
O6	0.0113 (18)	0.0200 (19)	0.0153 (17)	0.0061 (15)	0.0048 (14)	0.0015 (15)
O7	0.030 (2)	0.0118 (18)	0.026 (2)	−0.0036 (16)	0.0151 (18)	−0.0013 (16)
O8	0.0087 (17)	0.0261 (19)	0.0079 (16)	−0.0034 (15)	−0.0012 (14)	0.0016 (15)

### Geometric parameters (Å, º)

**Table:**

Pb1—O3^i^	2.504 (4)	P1—O3	1.536 (3)
Pb1—O8	2.539 (4)	P1—O4	1.559 (4)
Pb1—O6^ii^	2.614 (4)	P2—O5	1.514 (4)
Pb1—O4^i^	2.697 (4)	P2—O7	1.526 (4)
Pb1—O7^iii^	2.698 (4)	P2—O6	1.532 (4)
Pb1—O5	2.767 (4)	P2—O8	1.565 (4)
Pb1—O4^ii^	2.785 (4)	O1—Mn2^vi^	2.141 (4)
Mn1—O3^iv^	2.157 (3)	O2—Mn2^viii^	2.122 (4)
Mn1—O3^i^	2.157 (3)	O2—Mn2^ix^	2.208 (3)
Mn1—O6^ii^	2.168 (3)	O3—Mn1^ix^	2.157 (3)
Mn1—O6^v^	2.168 (3)	O3—Pb1^x^	2.504 (4)
Mn1—O1	2.195 (3)	O4—Pb1^x^	2.697 (4)
Mn1—O1^vi^	2.195 (3)	O4—Pb1^ii^	2.785 (4)
Mn2—O5^iii^	2.094 (4)	O5—Mn2^xi^	2.094 (4)
Mn2—O2^vii^	2.122 (4)	O6—Mn1^xii^	2.168 (3)
Mn2—O1^vi^	2.141 (4)	O6—Mn2^ii^	2.610 (4)
Mn2—O2^iv^	2.208 (3)	O6—Pb1^ii^	2.614 (4)
Mn2—O7^ii^	2.235 (4)	O7—Mn2^ii^	2.235 (4)
Mn2—O6^ii^	2.610 (4)	O7—Pb1^xi^	2.698 (4)
P1—O2	1.530 (3)	O8—H8	0.8600
P1—O1	1.531 (3)		
			
O3^i^—Pb1—O8	82.97 (11)	O3^i^—Mn1—O1^vi^	89.35 (13)
O3^i^—Pb1—O6^ii^	69.84 (11)	O6^ii^—Mn1—O1^vi^	81.84 (13)
O8—Pb1—O6^ii^	70.75 (11)	O6^v^—Mn1—O1^vi^	98.16 (13)
O3^i^—Pb1—O4^i^	56.56 (11)	O1—Mn1—O1^vi^	180.000 (1)
O8—Pb1—O4^i^	71.32 (11)	O5^iii^—Mn2—O2^vii^	100.04 (15)
O6^ii^—Pb1—O4^i^	116.47 (11)	O5^iii^—Mn2—O1^vi^	92.62 (15)
O3^i^—Pb1—O7^iii^	91.78 (12)	O2^vii^—Mn2—O1^vi^	129.62 (14)
O8—Pb1—O7^iii^	173.95 (12)	O5^iii^—Mn2—O2^iv^	176.09 (15)
O6^ii^—Pb1—O7^iii^	104.65 (12)	O2^vii^—Mn2—O2^iv^	79.73 (13)
O4^i^—Pb1—O7^iii^	108.25 (11)	O1^vi^—Mn2—O2^iv^	90.49 (14)
O3^i^—Pb1—O5	123.87 (12)	O5^iii^—Mn2—O7^ii^	87.34 (15)
O8—Pb1—O5	53.50 (11)	O2^vii^—Mn2—O7^ii^	96.38 (14)
O6^ii^—Pb1—O5	116.03 (11)	O1^vi^—Mn2—O7^ii^	133.01 (14)
O4^i^—Pb1—O5	75.23 (12)	O2^iv^—Mn2—O7^ii^	88.81 (14)
O7^iii^—Pb1—O5	132.49 (12)	O5^iii^—Mn2—O6^ii^	79.12 (14)
O3^i^—Pb1—O4^ii^	151.26 (10)	O2^vii^—Mn2—O6^ii^	157.01 (13)
O8—Pb1—O4^ii^	89.67 (11)	O1^vi^—Mn2—O6^ii^	73.21 (12)
O6^ii^—Pb1—O4^ii^	81.50 (10)	O2^iv^—Mn2—O6^ii^	99.54 (12)
O4^i^—Pb1—O4^ii^	145.63 (8)	O7^ii^—Mn2—O6^ii^	60.65 (12)
O7^iii^—Pb1—O4^ii^	93.55 (11)	O2—P1—O1	112.1 (2)
O5—Pb1—O4^ii^	70.46 (12)	O2—P1—O3	111.0 (2)
O3^iv^—Mn1—O3^i^	180.000 (1)	O1—P1—O3	108.4 (2)
O3^iv^—Mn1—O6^ii^	94.68 (14)	O2—P1—O4	109.9 (2)
O3^i^—Mn1—O6^ii^	85.32 (14)	O1—P1—O4	109.4 (2)
O3^iv^—Mn1—O6^v^	85.32 (14)	O3—P1—O4	105.9 (2)
O3^i^—Mn1—O6^v^	94.68 (14)	O5—P2—O7	113.5 (2)
O6^ii^—Mn1—O6^v^	180.00 (19)	O5—P2—O6	113.7 (2)
O3^iv^—Mn1—O1	89.35 (13)	O7—P2—O6	107.6 (2)
O3^i^—Mn1—O1	90.65 (13)	O5—P2—O8	102.2 (2)
O6^ii^—Mn1—O1	98.16 (13)	O7—P2—O8	109.8 (2)
O6^v^—Mn1—O1	81.84 (13)	O6—P2—O8	109.9 (2)
O3^iv^—Mn1—O1^vi^	90.65 (13)		

Symmetry codes: (i) *x*, −*y*+3/2, *z*+1/2; (ii) −*x*+1, −*y*+1, −*z*+1; (iii) −*x*+1, *y*−1/2, −*z*+3/2; (iv) −*x*, *y*−1/2, −*z*+1/2; (v) *x*−1, *y*, *z*; (vi) −*x*, −*y*+1, −*z*+1; (vii) *x*, −*y*+1/2, *z*+1/2; (viii) *x*, −*y*+1/2, *z*−1/2; (ix) −*x*, *y*+1/2, −*z*+1/2; (x) *x*, −*y*+3/2, *z*−1/2; (xi) −*x*+1, *y*+1/2, −*z*+3/2; (xii) *x*+1, *y*, *z*.

### Hydrogen-bond geometry (Å, º)

**Table:**

*D*—H···*A*	*D*—H	H···*A*	*D*···*A*	*D*—H···*A*
O8—H8···O4	0.86	1.60	2.437 (5)	164
O8—H8···O1	0.86	2.76	3.393 (5)	132
